# DNA methylation as an epigenetic biomarker in colorectal cancer

**DOI:** 10.3892/ol.2013.1606

**Published:** 2013-10-07

**Authors:** TIAGO DONIZETTI SILVA, VERONICA MARQUES VIDIGAL, ALEDSON VITOR FELIPE, JACQUELINE MIRANDA DE LIMA, RICARDO ARTIGIANI NETO, SARHAN SIDNEY SAAD, NORA MANOUKIAN FORONES

**Affiliations:** 1 Oncology Group, Gastroenterology Division, Federal University of São Paulo, São Paulo 04023-900, Brazil; 2 Department of Pathology, Federal University of São Paulo, São Paulo 04023-900, Brazil; 3 Department of Surgery, Federal University of São Paulo, São Paulo 04023-900, Brazil

**Keywords:** methylation, colorectal cancer, epigenetics

## Abstract

Sporadic colorectal cancer (CRC) is a consequence of the accumulation of genetic and epigenetic alterations that result in the transformation of normal colonic epithelial cells to adenocarcinomas. Studies have indicated that a common event in the tumorigenesis of CRC is the association of global hypomethylation with discrete hypermethylation at the promoter regions of specific genes that are involved in cell cycle regulation, DNA repair, apoptosis, angiogenesis, adhesion and invasion. The present study aimed to investigate the epigenetic changes (DNA methylation) in 24 candidate genes in CRC. A total of 10 candidate hypermethylated (HM) and unmethylated (UM) genes were identified that may be useful epigenetic markers for non-invasive CRC screening. The five genes that had the highest average UM percentages in the control group were *MLH1* (71.7%), *DKK2* (69.6%), *CDKN2A* (68.4%), *APC* (67.5%) and *hsa-mir-342* (67.4%). *RUNX3* (58.9%), *PCDH10* (55.5%), *SFRP5* (52.1%), *IGF2* (50.4%) and *Hnf1b* (50.0%) were the five genes with the highest average HM percentages in the test group. In summary, the present preliminary study identified the methylation profiles of normal and cancerous colonic epithelial tissues, and provided the groundwork for future large-scale methylation studies.

## Introduction

Colorectal cancer (CRC) is the third and fourth most common cancer in females and males, respectively, accounting for 610,000 mortalities worldwide each year. An estimated 14,180 and 15,960 new cases of CRC in males and females, respectively, occurred in Brazil in 2012. These values correspond with an estimated risk of 15 new CRC cases in males and 16 in females per 100,000 per year (1).

The overall survival rate of patients with CRC is highly dependent on the disease stage at the time of diagnosis. The estimated five-year survival rates range from 85–90% for patients with stage I tumors to <5% for patients with stage IV diseases. Although the number of new CRC cases and mortalities from CRC has declined in more recent years, approximately half of all CRC patients develop a local recurrence or distant metastases during the course of their disease (2). To date, clinical, pathological or molecular markers for the identification of patients who are at risk of developing distant metastases have not been established (3).

CRC is curable in ~90% cases if it is detected at an early stage (4). The early detection of CRC through screening programs that detect mucosal changes which are predictive of colorectal tumors reduces the incidence and mortality rates of this disease (5,6). Current non-invasive screening procedures for CRC are not effective. Fecal occult blood test (FOBT), a commonly used non-invasive screening procedure, reduces CRC-related mortality by 20% when performed every two years (7). Despite improvements in sensitivity, FOBT has a low detection rate for early-stage tumors and precancerous lesions, such as polyps (8). Although invasive screening tests, including colonoscopy and retosigmoidoscopia are more effective, they are extremely costly and require extensive preparation of the bowel, invasion of patient privacy and sedation (9). Therefore, there is a requirement for sensitive and specific diagnostic markers that may be used to control the adenoma-to-carcinoma sequence of CRC (2).

Sporadic CRC is a consequence of the accumulation of genetic and epigenetic alterations that result in the transformation of normal colonic epithelial cells to adenocarcinomas. The loss of genomic stability and subsequent genetic alterations in tumor suppressor genes and oncogenes initiate carcinogenesis and tumor progression (10). CRC carcinogenesis is associated with alterations in oncogenes, including *KRAS*, and tumor suppressor genes, including *adenomatous polyposis coli* (*APC*), deleted in CRC and *tumor protein p53*. Over 25 years ago, Vogelstein *et al* identified an extensive loss of DNA methylation in the non-promoter regions in colon cancer cells. This global hypomethylation has been associated with an increased genomic instability and overexpression of a variety of genes that are implicated in CRC pathogenesis (11). A common event in the tumorigenesis of CRC is believed to be the association of global hypomethylation with discrete hypermethylation at the promoter regions of specific genes that are involved in cell cycle regulation, DNA repair, apoptosis, angiogenesis, adhesion and invasion (12). As the aberrant methylation of promoter regions precedes genetic alterations, epigenetic events that are associated with CRC may have great potential to be used as biomarkers for the detection of early-stage disease (13).

The aim of the present study was to investigate the epigenetic changes (DNA methylation) in 24 candidate genes in CRC tumors. A total of five candidate hypermethylated (HM) genes were identified, which may be useful epigenetic markers for non-invasive CRC screening.

## Materials and methods

### Subjects

The epigenetic changes in 24 candidate genes (Table I) were evaluated in tissues from patients with CRC and from normal controls. The test group consisted of 10 randomly selected patients with primary colorectal adenocarcinoma who underwent surgical resection at the Federal University of São Paulo (São Paulo, Brazil). The control group consisted of 10 individuals with a normal colonoscopy and without a previous diagnosis of inflammatory bowel disease or malignant disease. This study was approved by the Ethical Committee of the Federal of São Paulo (São Paulo, Brazil). All patients provided written informed consent.

### DNA extraction

The CRC tissues were removed by the surgical pathologist and immediately frozen in liquid nitrogen. The freshly frozen tumor tissues (25 mg) were cut into small sections and incubated for 6 h at 56ºC. During the incubation period, the tissue samples were vortexed every 30 min to promote lysis. Biopsy specimens were collected from the control group during the colonoscopy and placed into tubes containing Allprotect (Qiagen, Hilden, Germany). Sterile gauze was used to remove the excess Allprotect from the specimens. The entire biopsy fragment (≤10 mg) was used for DNA extraction. The biopsy fragments were incubated overnight at 56ºC and periodically vortexed to promote lysis. The DNA was extracted from the surgical and biopsy specimens using the QIAamp DNA Mini kit (Qiagen) and QIAamp DNA Micro kit (Qiagen), respectively, according to the manufacturer’s instructions. The DNA was eluted in nuclease-free water and stored at −20ºC. The extracted DNA was quantified using a NanoDrop 1000 spectrophotometer (Thermo Fisher Scientific Inc., Wilmington, DE, USA).

### Methylation analysis

The methylation analysis was performed using the Methyl-Profiler™ DNA Methylation Polymerase Chain Reaction (PCR) Array System (SA Biosciences, Hilden, Germany). The Methyl-Profiler PCR Array System relies on the differential cleavage of target sequences using two separate restriction endonucleases, whose activities require either the presence or absence of methylated cytosines in their respective recognition sequences. The relative amount of DNA that remained following each enzyme digestion was quantified by quantitative PCR (qPCR) using the ABI StepOnePlus™ RT-PCR System (Applied Biosystems, Carlsbad, CA, USA).

The relative fractions of HM, intermediate methylated and unmethylated (UM) DNA were determined by comparing the amount in each digestion with that of a mock digest using the standard ΔCt method.

### Statistical analysis

Receiver operating characteristic curves were used to assess the sensitivity, specificity and accuracy of the cancer detection methods, and for the prediction of the cancer genes. Non-parametric tests were used for the statistical analysis due to the low subject numbers (<25 subjects). The Wilcoxon-Mann-Whitney test was used to compare the HM and UM genes in the test and control groups. A multivariate cluster analysis was performed using Euclidean distance to group the genes that displayed similar methylation statuses. P<0.05 was considered to indicate a statistically significant difference, and P-values of 0.05–0.10 were considered marginally significant. The statistical analyses were performed using SPSS software, version 16 (SPSS, Inc., Chicago, IL, USA), Minitab 15 (Minitab, State College, PA, USA) and Excel Office 2007 (Microsoft, Redmond, WA, USA) (14–15).

## Results

The present study identified five genes among a panel of 24 cancer-related genes, which had the greatest potential to be CRC biomarkers based on their epigenetic alterations. From the test and control groups, one patient each was excluded due to technical issues. Therefore, nine patients were assigned to the test and control groups, respectively. The methylation statuses of the 24 genes from the test and control groups are shown in Tables I and II.

The five genes that had the highest average UM percentages in the control group were *MLH1* (71.7%), *DKK2* (69.6%), *CDKN2A* (68.4%), *APC* (67.5%) and *hsa-mir-342* (67.4%; Table III). *RUNX3* (58.9%), *PCDH10* (55.5%), *SFRP5* (52.1%), *IGF2* (50.4%) and *Hnf1b* (50.0%) were the five genes with the highest average HM percentages in the test group (Table IV).

**Figure 1:**
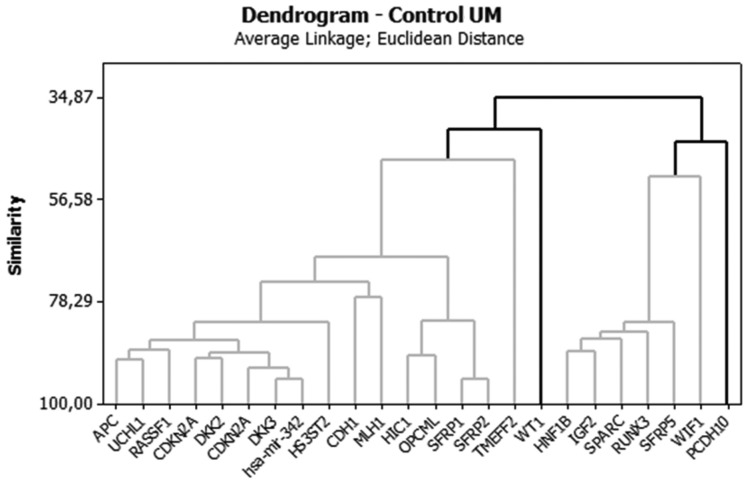
Hierarchical clustering diagram showing the UM genes in the control group separated into two groups, with extensive UM genes that are compatible with the other groups that demonstrate low levels of methylation. Bold line depicts high levels of UM genes in the control group. UM, unmethylated.

**Figure 2:**
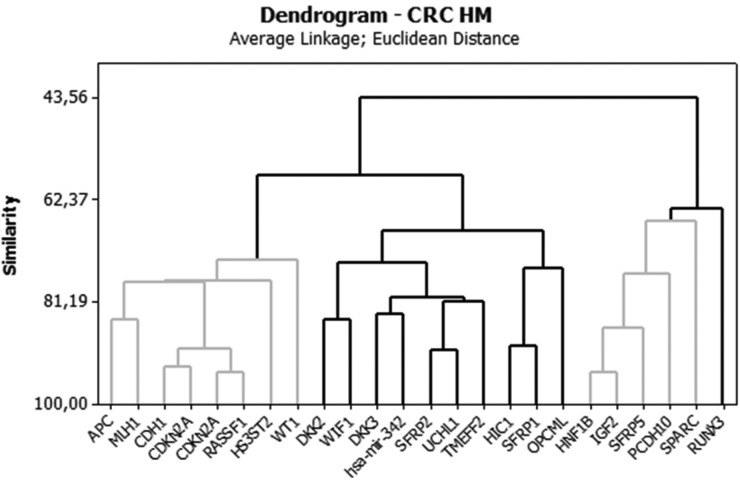
Hierarchical clustering diagram showing the HM genes in the cancer group, which are clustered into into three groups, which correspond to having extensive methylation compatible with the other groups demonstrating low levels of methylation. Bold line depicts high levels of hypermethylation in the cancer group. CRC, colorectal cancer; HM, hypermethylated.

The analysis of groups or clusters is an exploratory multivariate analysis technique that allows subjects to be grouped into homogeneous or compact groups based on one or more common characteristics (14,15). Each subject in the same cluster is more similar to each other than to those in the other clusters. In the present study, a cluster analysis using Euclidean distance was performed in order to group the genes that displayed similar methylation behaviors. Tables V and VI show the distance values between the centers (centroids) of the clusters for each analysis. The larger the distance between the clusters, the more distinct the clusters are. Cluster analysis data are best visualized using graphs called dendograms, which display the associations between the clusters (i.e., groups of genes). (Figs. 1 and 2).

## Discussion

Epigenetics is the study of heritable and age-related modifications of the genome that occur without a change in the primary DNA sequence. In recent years, epigenetics has become an emerging field due to the fundamental role of epigenetic modifications, including DNA methylation, specific histone modifications and noncoding RNAs (i.e., silencing RNA and microRNA), in the regulation of gene expression (16). Epigenetic alterations, particularly aberrant DNA methylation, contribute to tumor initiation and progression. The methylation of tumor-specific loci, rather than the presence of methylation, is key in carcinogenesis (2). The finding that aberrant DNA methylation is associated with the occurrence of early CRC lesions suggests that epigenetic alterations are involved in the initiation of CRC. However, the possibility that aberrant DNA methylation is a secondary phenomenon cannot be excluded (17). Therefore, a knowledge of DNA methylation patterns and the detection of HM genes in normal and cancerous tissues may facilitate an understanding of the tumorigenesis of CRC, leading to the identification of new diagnostic, prognostic and predictive biomarkers. Furthermore, the epigenetic changes due to DNA methylation in cancer represent an attractive therapeutic target, as they are reversible in nature, unlike genetic alterations (18). Since methylated genes that are present in tumor tissues may be identified in urine and serum, epigenetic biomarkers represent a non-invasive screening method for CRC diagnosis.

The methylation of CpG islands occurs early in carcinogenesis but may also be detected in normal epithelium as a result of aging and inflammation. As methylated alleles may be detected with a very high degree of sensitivity, there is great scope in using methylation as a potential early detection system for cancer. A variety of genome-wide methods are currently available for the discovery of differentially methylated markers. However, these methods typically produce large numbers of potential candidates. An estimated 500 genes may be involved in CRC based on DNA methylation studies (19). Thus, downstream selection processes are critical for the identification of clinically relevant markers that have the necessary properties to perform adequately in future tests (20).

Despite the association of epigenetic alterations in DNA methylation and carcinogenesis, certain studies have failed to demonstrate an association between the methylation status of a gene and cancer (21). Furthermore, certain studies have indicated that methylated genes retain their normal function (21). Based on this information, it is important to determine not only the presence of gene methylation but also the extent of methylation. For example, a gene that is 30% methylated may display alternative behaviors than a gene that is 60% methylated. Based only on the presence of methylation, the two genes would have been classified in the same group. Numerous studies use qualitative techniques to assess methylation status by defining a cut-off value based on the amount of methylated cytosines that are required to repress gene expression. Based on a common PCR-based method of methylation analysis using bisulfite treatment of DNA, the minimum methylation level for a gene to be considered HM is 10–20% methylation (22). In the present study, using a qPCR-based technique, a group of five HM genes with the highest percentage of methylation were identified in CRC patients, *RUNX3*, *PCDH10*, *SFRP5*, *IGF2* and *Hnf1b*. These genes were observed to have the greatest potential of gene expression repression and, therefore, were the most promising biomarkers for the diagnosis of CRC. A group of five genes that had the highest unmethylation percentage were identified in the control group, *MLH1*, *DKK2*, *CDKN2A*, *APC* and *hsa-mir-342*. Alterations in these genes are commonly associated with CRC carcinogenesis. These 10 genes did not differ quantitatively between the test and control groups, but they qualitatively represented the genes with the highest percentages of methylation and unmethylation. These data suggested that in the control group, the genes were not providing a protective effect, but in the carcinogenic process, they submitted a contrary profile.

In summary, the present preliminary study identified the methylation profiles of normal and cancerous colonic epithelial tissues, and provided the groundwork for future large-scale methylation studies. As DNA methylation is significant in CRC initiation, this study will be useful in understanding the epigenetic mechanisms of CRC and identifying biomarkers for the detection of early-stage disease.

## Figures and Tables

**Table I:** HM and UM genes in the control group.

Control	Mean (%)	Median (%)	SD (%)	N	CI (%)	P-value
*APC*						
HM	32.5	34.0	17.6	9	11.5	0.028
UM	67.5	66.0	17.6	9	11.5	
*CDH1*						
HM	34.6	39.4	17.6	9	11.5	0.043
UM	65.4	60.6	17.6	9	11.5	
*CDKN2A*						
HM	31.6	22.3	17.7	9	11.5	0.028
UM	68.4	77.7	17.7	9	11.5	
*CDKN2A*						
HM	33.9	27.6	15.8	9	10.4	0.043
UM	66.1	72.4	15.8	9	10.4	
*DKK2*						
HM	30.4	25.0	18.9	9	12.4	0.028
UM	69.6	75.0	18.9	9	12.4	
*DKK3*						
HM	33.7	29.8	17.0	9	11.1	0.046
UM	66.3	70.2	17.0	9	11.1	
*HIC1*						
HM	40.9	42.8	10.0	9	6.5	0.043
UM	59.1	57.2	10.0	9	6.5	
*HNF1B*						
HM	50.0	50.0	0.0	9	-	1.000
UM	50.0	50.0	0.0	9	-	
*HS3ST2*						
HM	32.8	25.6	17.4	9	11.4	0.046
UM	67.2	74.4	17.4	9	11.4	
*IGF2*						
HM	51.7	50.0	4.1	8	2.9	0.465
UM	48.3	50.0	4.1	8	2.9	
*MLH1*						
HM	28.9	26.9	20.6	9	13.5	0.018
UM	71.1	73.1	20.6	9	13.5	
*hsa-mir-342*						
HM	32.6	30.1	17.4	9	11.4	0.028
UM	67.4	69.9	17.4	9	11.4	
*OPCML*						
HM	42.7	47.6	10.3	9	6.7	0.043
UM	57.3	52.4	10.3	9	6.7	
*PCDH10*						
HM	47.7	50.0	18.6	9	12.2	0.612
UM	52.3	50.0	18.6	9	12.2	
*RASSF1*						
HM	32.5	33.4	16.6	9	10.8	0.028
UM	67.5	66.6	16.6	9	10.8	
*RUNX3*						
HM	52.6	50.0	4.3	9	2.8	0.068
UM	47.4	50.0	4.3	9	2.8	
*SFRP1*						
HM	38.2	33.8	11.9	9	7.8	0.043
UM	61.8	66.2	11.9	9	7.8	
*SFRP2*						
HM	37.4	35.4	13.1	9	8.6	0.043
UM	62.6	64.6	13.1	9	8.6	
*SFRP5*						
HM	52.5	50.0	5.2	9	3.4	0.180
UM	47.5	50.0	5.2	9	3.4	
*SPARC*						
HM	48.7	50.0	2.3	9	1.5	0.593
UM	49.2	50.0	4.4	9	2.9	
*TMEFF2*						
HM	29.2	22.7	17.4	9	11.3	0.046
UM	63.6	77.3	29.4	9	19.2	
*UCHL1*						
HM	34.5	37.8	16.2	9	10.6	0.043
UM	65.5	62.2	16.2	9	10.6	
*WIF1*						
HM	34.8	50.0	19.0	9	12.4	0.080
UM	53.1	50.0	17.7	9	11.6	
*WT1*						
HM	28.3	14.0	26.2	9	17.1	0.069
UM	65.1	54.1	27.3	9	17.8	

-, not applicable for the statistics. CI, confidence interval; SD, standard deviation; APC, adenomatous polyposis coli; HM, hypermethylated; UM, unmethylated.

**Table II:** HM and UM genes in the CRC group.

CRC	Mean (%)	Median (%)	SD (%)	N	CI (%)	P-value
*APC*						
HM	33.3	37.6	16.9	9	11.1	0.012
UM	66.7	62.4	16.9	9	11.1	
*CDH1*						
HM	36.5	47.0	14.4	9	9.4	0.018
UM	63.5	53.0	14.4	9	9.4	
*CDKN2A*						
HM	36.1	43.8	14.7	9	9.6	0.028
UM	63.9	56.2	14.7	9	9.6	
*CDKN2A*						
HM	37.7	46.0	15.0	9	9.8	0.046
UM	62.3	54.0	15.0	9	9.8	
*DKK2*						
HM	44.8	50.0	20.9	8	14.5	0.500
UM	55.2	50.0	20.9	8	14.5	
*DKK3*						
HM	38.7	39.8	20.4	9	13.3	0.116
UM	61.3	60.2	20.4	9	13.3	
*HIC1*						
HM	44.9	50.0	11.2	9	7.3	0.345
UM	55.1	50.0	11.2	9	7.3	
*HNF1B*						
HM	50.0	50.0	0.0	9	-	1.000
UM	50.0	50.0	0.0	9	-	
*HS3ST2*						
HM	37.0	44.5	15.5	8	10.7	0.043
UM	63.0	55.5	15.5	8	10.7	
*IGF2*						
HM	50.4	50.0	3.3	5	2.9	0.593
UM	49.6	50.0	3.3	5	2.9	
*MLH1*						
HM	33.6	39.5	18.1	9	11.8	0.018
UM	65.0	56.2	19.0	9	12.4	
*hsa-mir-342*						
HM	38.8	46.9	21.5	8	14.9	0.172
UM	61.2	53.1	21.5	8	14.9	
*OPCML*						
HM	46.8	47.0	14.5	9	9.5	0.446
UM	52.5	50.0	14.7	9	9.6	
*PCDH10*						
HM	55.5	50.0	9.2	9	6.0	0.144
UM	44.3	50.0	9.0	9	5.9	
*RASSF1*						
HM	34.9	37.6	14.8	9	9.7	0.028
UM	61.5	56.7	15.3	9	10.0	
*RUNX3*						
HM	58.9	53.8	13.4	9	8.8	0.063
UM	41.1	46.2	13.4	9	8.8	
*SFRP1*						
HM	47.0	50.0	9.5	9	6.2	0.686
UM	53.0	50.0	9.5	9	6.2	
*SFRP2*						
HM	44.5	50.0	16.3	9	10.7	0.498
UM	55.5	50.0	16.3	9	10.7	
*SFRP5*						
HM	52.1	50.0	5.4	9	3.5	0.225
UM	47.9	50.0	5.4	9	3.5	
*SPARC*						
HM	47.9	50.0	13.6	9	8.9	0.345
UM	52.1	50.0	13.6	9	8.9	
*TMEFF2*						
HM	41.2	47.0	21.9	9	14.3	0.327
UM	54.6	50.0	22.1	9	14.5	
*UCHL1*						
HM	43.2	47.2	18.9	9	12.4	0.271
UM	56.8	52.8	18.9	9	12.4	
*WIF1*						
HM	46.1	50.0	20.7	9	13.5	0.866
UM	49.6	48.6	19.4	9	12.7	
*WT1*						
HM	29.8	26.6	17.8	9	11.6	0.017
UM	70.2	73.4	17.8	9	11.6	

-, not applicable for the statistics. CRC, colorectal cancer; CI, confidence interval; SD, standard deviation; APC, adenomatous polyposis coli; HM, hypermethylated; UM, unmethylated.

**Table III:** UM symbol in the control group.

UM control	Mean (%)	Median (%)	SD (%)	N	CI (%)	P-value
*APC*	67.5	66.0	17.6	9	11.5	0.688
*CDH1*	65.4	60.6	17.6	9	11.5	0.393
*CDKN2A*	68.4	77.7	17.7	9	11.5	0.448
*CDKN2A*	66.1	72.4	15.8	9	10.4	0.301
*DKK2*	69.6	75.0	18.9	9	12.4	0.824
*DKK3*	66.3	70.2	17.0	9	11.1	0.305
*HIC1*	59.1	57.2	10.0	9	6.5	0.163
*HNF1B*	50.0	50.0	0.0	9	-	0.002
*HS3ST2*	67.2	74.4	17.4	9	11.4	0.349
*IGF2*	48.3	50.0	4.1	8	2.9	0.008
*MLH1*	71.1	73.1	20.6	9	13.5	-
*hsa-mir-342*	67.4	69.9	17.4	9	11.4	0.562
*OPCML*	57.3	52.4	10.3	9	6.7	0.163
*PCDH10*	52.3	50.0	18.6	9	12.2	0.037
*RASSF1*	67.5	66.6	16.6	9	10.8	0.451
*RUNX3*	47.4	50.0	4.3	9	2.8	0.001
*SFRP1*	61.8	66.2	11.9	9	7.8	0.163
*SFRP2*	62.6	64.6	13.1	9	8.6	0.225
*SFRP5*	47.5	50.0	5.2	9	3.4	0.002
*SPARC*	49.2	50.0	4.4	9	2.9	0.011
*TMEFF2*	63.6	77.3	29.4	9	19.2	0.448
*UCHL1*	65.5	62.2	16.2	9	10.6	0.345
*WIF1*	53.1	50.0	17.7	9	11.6	0.022
*WT1*	65.1	54.1	27.3	9	17.8	0.626

-, not applicable for the statistics. UM, unmethylated; APC, adenomatous polyposis coli; CI, confidence interval; SD, standard deviation.

**Table IV:** HM Symbol in the CRC group.

HM CRC	Mean (%)	Median (%)	SD (%)	N	CI (%)	P-value
*APC*	33.3	37.6	16.9	9	11.1	0.001
*CDH1*	36.5	47.0	14.4	9	9.4	0.003
*CDKN2A*	36.1	43.8	14.7	9	9.6	0.004
*CDKN2A*	37.7	46.0	15.0	9	9.8	0.008
*DKK2*	44.8	50.0	20.9	8	14.5	0.098
*DKK3*	38.7	39.8	20.4	9	13.3	0.018
*HIC1*	44.9	50.0	11.2	9	7.3	0.035
*HNF1B*	50.0	50.0	0.0	9	-	0.024
*HS3ST2*	37.0	44.5	15.5	8	10.7	0.005
*IGF2*	50.4	50.0	3.3	5	2.9	0.200
*MLH1*	33.6	39.5	18.1	9	11.8	0.002
*hsa-mir-342*	38.8	46.9	21.5	8	14.9	0.053
*OPCML*	46.8	47.0	14.5	9	9.5	0.056
*PCDH10*	55.5	50.0	9.2	9	6.0	0.387
*RASSF1*	34.9	37.6	14.8	9	9.7	0.003
*RUNX3*	58.9	53.8	13.4	9	8.8	-
*SFRP1*	47.0	50.0	9.5	9	6.2	0.065
*SFRP2*	44.5	50.0	16.3	9	10.7	0.053
*SFRP5*	52.1	50.0	5.4	9	3.5	0.225
*SPARC*	47.9	50.0	13.6	9	8.9	0.067
*TMEFF2*	41.2	47.0	21.9	9	14.3	0.057
*UCHL1*	43.2	47.2	18.9	9	12.4	0.046
*WIF1*	46.1	50.0	20.7	9	13.5	0.120
*WT1*	29.8	26.6	17.8	9	11.6	0.003

-, not applicable for the statistics. HM, hypermethylated; CRC, colorectal cancer; APC, adenomatous polyposis coli; CI, confidence interval; SD, standard deviation.

**Table VI:** Distance of centers of clusters in CRC patients.

CRC	Cluster 1	Cluster 2	Cluster 3
HM			
Cluster 2	0.4219		
Cluster 3	0.6637	0.4780	
Cluster 4	1.0034	0.7238	0.3792
UM			
Cluster 2	0.4585		
Cluster 3	0.3655	0.3473	
Cluster 4	0.7114	0.5155	0.4690

CRC, colorectal cancer; HM, hypermethylated; UM, unmethylated.

**Table V:** Distance of centers of clusters in control.

Control	Cluster 1	Cluster 2	Cluster 3
HM			
Cluster 2	0.6463		
Cluster 3	0.6127	0.5009	
Cluster 4	0.4068	0.9848	0.8757
UM			
Cluster 2	0.6480		
Cluster 3	0.6076	0.5782	
Cluster 4	0.5948	0.8421	1.0852

HM, hypermethylated; UM, unmethylated.

## References

[1] INCA (2013). Instituto Nacional do Câncer.

[2] Kim MS, Lee J, Sidransky D (2010). DNA methylation markers in colorectal cancer. Cancer Metastasis Rev.

[3] Pellegrini ML, Argibay P, Gómez DE (2011). Genetics and epigenetics of colorectal cancer. Acta Gastroenterol Latinoam.

[4] Toribara NW, Sleisenger MH (1995). Screening for colorectal cancer. N Eng J Med.

[5] Winawer SJ (2007). Colorectal cancer screening. Best Pract Res Clin Gastroenterol.

[6] Pignone MP, Lewis CL (2009). Using quality improvement techniques to increase colon cancer screening. Am J Med.

[7] Atkin W (2003). Options for screening for colorectal cancer. Scand J Gastroenterol Suppl.

[8] Imperiale TF, Ransohoff DF, Itzkowitz SH (2004). Fecal DNA versus fecal occult blood for colorectal-cancer screening in an average-risk population. N Engl J Med.

[9] Grützmann R, Molnar B, Pilarsky C (2008). Sensitive detection of colorectal cancer in peripheral blood by septin 9 DNA methylation assay. PLoS One.

[10] Grady WM, Carethers JM (2008). Genomic and epigenetic instability in colorectal cancer pathogenesis. Gastroenterology.

[11] Vogelstein B, Fearon ER, Hamilton SR (1988). Genetic alterations during colorectal-tumor development. N Eng J Med.

[12] Carmona FJ, Esteller M (2010). Epigenomics of human colon cancer. Mutat Res.

[13] Rawson JB, Bapat B (2012). Epigenetic biomarkers in colorectal cancer diagnostics. Expert Rev Mol Diagn.

[14] Conover WI (1971). Practcal Nonparametric Statistics.

[15] Daniel WW, Cross CL (1995). Biostatistics: a foundation for analysis in the health sciences.

[16] Migheli F, Migliore L (2012). Epigenetics of colorectal cancer. Clin Genet.

[17] Lange CP, Campan M, Hinoue T (2012). Genome-scale discovery of DNA-methylation biomarkers for blood-based detection of colorectal cancer. PLoS One.

[18] Karpf AR, Jones DA (2012). Reactivating the expression of methylation silenced genes in human cancer. Oncogene.

[19] Schuebel KE, Chen W, Cope L (2007). Comparing the DNA hypermethylome with gene mutations in human colorectal cancer. PLoS Genet.

[20] Lofton-Day C, Model F, Devos T (2008). DNA methylation biomarkers for blood-based colorectal cancer screening. Clin Chem.

[21] Goel A, Boland CR (2012). Epigenetics of colorectal cancer. Gastroenterology.

[22] Ausch C, Kim YH, Tsuchiya KD (2009). Comparative analysis of PCR-based biomarker assay methods for colorectal polyp detection from fecal DNA. Clin Chem.

